# The Crucial Role of Epigenetic Modifications in Wharton’s Jelly Stem Cells

**DOI:** 10.3390/ijms26157169

**Published:** 2025-07-24

**Authors:** Mao Yang, Juan Wang, Wensheng Deng, Qiang Wu

**Affiliations:** 1 The State Key Laboratory of Quality Research in Chinese Medicine, Macau University of Science and Technology, Macao 999078, Chinawangjuan@wust.edu.cn (J.W.); 2 School of Life Science and Health, Wuhan University of Science and Technology, Wuhan 430065, China

**Keywords:** Warton’s jelly stem cells, differentiation, DNA methylation, histone modifications, non-coding RNAs, miRNAs

## Abstract

Wharton’s jelly mesenchymal stem cells (WJ-SCs) are a promising source for regenerative medicine due to their multipotency, low immunogenicity, and ethical acceptability. Epigenetic regulation plays a crucial role in modulating their proliferation, differentiation, and therapeutic potential. Key mechanisms, including DNA methylation, histone modifications, and non-coding RNAs (e.g., miRNAs and lncRNAs), influence WJ-SC behavior by dynamically altering gene expression without changing the DNA sequence. DNA methylation often silences genes involved in differentiation, while histone acetylation/methylation can activate or repress lineage-specific pathways. Non-coding RNAs further fine-tune these processes by post-transcriptional regulation. Understanding these mechanisms could optimize WJ-SC-based therapies for tissue repair and immune modulation. This review summarizes current insights into epigenetic regulation in WJ-SCs and its implications for regenerative applications.

## 1. Introduction

Stem cell therapy offers new hope for treating many diseases. Embryonic stem cells (ESCs) and induced pluripotent stem cells (iPSCs) are currently being used in research in the field of cell therapy and regenerative medicine to potentially replace damaged or diseased tissues. However, ESCs present sensitive ethical concerns in clinical applications and production [1]. iPSCs have an increased risk of abnormalities in genetic material due to high mitotic frequency during reprogramming, cloning, culture, and differentiation, which can lead to tumorigenicity. Possible mechanisms include DNA sequence alterations, epigenetic modifications, and chromosomal abnormalities [2,3,4]. Therefore, it is crucial to explore alternative sources of stem cells.

Mesenchymal stem cells (MSCs) are multi-potent progenitor cells predominantly found in bone marrow and were first isolated and analyzed by Alexander Friedenstein in the late 1960s [5]. MSCs are morphologically identical in appearance to fibroblasts and possess a strong self-renewal capacity, maintaining their ability to form stromal tissues specific to corresponding hematopoietic organs, even after successive passages in vitro [6]. MSCs can be derived from various sources, including adipose tissue, tendons, membranes, lungs, and periodontal ligaments. They can also be isolated from fetal tissues such as umbilical cord blood, umbilical cord stroma, placenta, and amniotic fluid [7,8,9]. In addition, MSCs are easy to proliferate and do not cause teratomas [10].

Wharton’s Jelly stem cells (WJ-SCs) are derived from umbilical cords. WJ-SCs expresses MSC phenotype-defining cell surface markers including CD90, CD73, and CD105 [11]. However, WJ-SCs display distinct gene expression profiles compared to human ESCs and other types of MSCs [12,13]. Importantly, WJ-SCs are a more primitive cell population with a faster multiplication time and possess a higher number of senescent passages compared to bone marrow MSCs [14]. In addition, WJ-SCs have lower immunogenicity and are less likely to cause post-transplantation rejection reactions with their host [15]. Therefore, WJ-SCs are a promising alternative to bone marrow mesenchymal stem cells (BM-MSCs) and adipose-derived stem cells (AD-SCs) due to their neonatal origin with less DNA damage, high proliferation rate, strong immunomodulation, and non-invasive sourcing [16,17,18]. Moreover, they are free from the ethical controversies associated with ESCs and do not carry the tumor risks linked to iPSCs or ESCs (Table 1). Given these advantages possessed by WJ-SCs, more and more studies have begun to focus on their genetic/epigenetic regulation and clinical applications. Although the genetic material of stem cells is DNA, their behavior is regulated at multiple levels, one of which includes epigenetic regulatory processes. Epigenetics has been defined as gene expression changes caused by alterations in chromatin status in the absence of changes in DNA sequence, such as DNA methylation and histone modifications [19].

## 2. Epigenetic Modifications in Cell Fate Decision of WJ-SCs

### 2.1. DNA Methylation

DNA methylation regulates cell stemness, differentiation and senescence by modulating chromosomal properties. DNA methylation is catalyzed by the DNA methyltransferase (DNMT) family, which transfers methyl groups from S-adenosylmethionine (SAM), the methyl donor, to the fifth carbon of CpG cytosine residues to form 5-methylcytosine (5mC) [20]. DNA plays a crucial role in gene regulation, genomic imprinting, X-chromosome inactivation, and suppression of transposable elements. Dysregulation of DNMTs is linked to cancer (hypermethylation of tumor suppressor genes), neurodevelopmental disorders, and autoimmune diseases. DNMTs are classified into two main categories based on their function: DNMT1 is primarily responsible for DNA methylation maintenance [21], while DNMT3a and DNMT3b are de novo methyltransferases that establish new methylation patterns for unmodified DNA [22]. Though how DNMTs regulate gene expression and other cellular activities in WJ-SCs are still understudied, the functions of DNMTs in ESCs and BM-MSCs have been extensively investigated, and the gained knowledge may shed light on understanding their roles in WJ-SCs.

DNMT1 is the primary maintenance methyltransferase that copies methylation patterns during DNA replication, ensuring epigenetic inheritance [23]. DNMT1 is essential for stem cell maintenance and loss of DNMT1 leads to global hypomethylation and embryonic lethality [23,24]. In WJ-SCs, DNMT1 is essential for methylation maintenance at promoters of pluripotency genes *OCT4* and *NANOG* to prevent spontaneous differentiation [25]. In turn, OCT4 and NANOG can directly regulate Dnmt1 to maintain self-renewal and undifferentiated state in MSCs [26]. DNMT1 is crucial for the process of osteogenic differentiation of MSCs [27].

DNMT3A/3B: the DNMT3 family members DNMT3A, DNMT3B and DNMT3L are de novo methyltransferases [28]. DNMT3L can bind to both DNMT3A and DNMT3B and enhance their catalytic activity [29]. DNMT3A establishes new methylation patterns, particularly at unmethylated CpG sites [30]. DNMT3B is responsible for methylation of CpG-poor regions, including LINEs (Long Interspersed Nuclear Elements), SINEs (Short Interspersed Nuclear Elements), and gene bodies [30,31]. DNMT3B plays an important role in chondrogenesis and genome instability [32]. DNMT3L is critical for X-chromosome inactivation and genomic imprinting [33].

DNMT expression is greatly reduced when cells reach terminal differentiation. The phosphatidylinositol 3-kinase (PI3K)/Akt pathway is activated in response to a variety of endogenous and exogenous stimuli to block cell death induced by multiple apoptotic signals [34]. Activation of this pathway upregulates DNMT1 and DNMT3b, with DNMT1 partially suppressing p53 by binding to its promoter site [35]. The main function of the EID (E1A-like inhibitor of differentiation) gene family is to act as a p300/CBP repressor in response to cellular transformation, growth arrest, or apoptosis [36]. During WJ-SC differentiation, EID3 modulates differentiation by directly interacting with DNMT3A [37]. Additionally, the transcription factors OCT4 and NANOG were previously identified as transcription factors controlling *DNMT1*, regulate DNMT1 expression by binding to its promoter, thereby inhibiting WJ-SC differentiation [38].

Demethylation is mediated by the TET family of demethylases TET1, TET2 and TET3, which convert 5-methylcytosine (5-mC) to 5-hydroxymethylcytosine (5-hmC). The oxidized derivatives through Fe (II)- and α-ketoglutarate (α-KG)-dependent reactions can promote DNA demethylation and gene transcription [39]. In WJ-SCs, both TET1 and TET2 are associated with proliferative capacity, and their reduced expression can lead to a significant reduction in cell proliferation [40].

Treatment of WJ-MSCs with DNMT inhibitors like 5-azacytidine has been shown to globally erase DNA methylation marks and induce cardiomyogenic differentiation [41]. Notably, 5-azacytidine treatment enhances the immunomodulatory properties of WJ-MSCs, resulting in greater suppression of monocyte proliferation and reduced migration toward activated T cells. This effect is mediated through two mechanisms: (1) activation of immunomodulatory factor promoters (COX2 and PTGES) and (2) hypomethylation of migration-associated genes (CXCR2 and CXCR4) [42].

Downregulation of DNMT1 may lead to overexpression of *OCT4* and *c-MYC*, both of which can subsequently enhance the expression of the *SIRT1* gene [43]. SIRT1, a class III histone deacetylase, plays a key role in gene regulation, genome stability, apoptosis, autophagy, senescence, and tumorigenesis. The hypomethylated state of *GATA4*, *NKX2.5*, *MEF2C*, and *TBX5* promotes the differentiation of WJ-SCs into cardiomyocytes. Additionally, CpG island methylation dynamically regulates gene expression in WJ-SCs, with genes harboring low levels of methyl modifications on promoter CGIs exhibiting higher expression levels than those with hypermethylated promoters. Notably, hypermethylation of homeobox genes drives WJ-MSC skeletal lineage commitment.

Furthermore, the *ITIH5* (inter-alpha (globin) inhibitor H5) and *FGF21* (fibroblast growth factor 21) genes are associated with lipid metabolism [44]. Hypomethylation of the CpG site Cg00221794, located near the *HS1BP3* (hematopoietic cell-specific Lyn substrate 1-binding protein 3) gene, significantly upregulates *HS1BP3* gene and modulates autophagy in WJ-SCs [45]. Similarly, hypomethylation of the *PGC-1α* promoter enhances myogenic differentiation potential and mitochondrial function in WJ-MSCs [46].

Finally, the long non-coding RNA CIR interacts with EZH2, facilitating EZH2-mediated H3K27 trimethylation of ATOH8 to suppress chondrogenic differentiation in WJ-SCs [47].

### 2.2. Histone Modifications

Histones are fundamental components of nucleosomes, and their post-translational modifications (PTMs) play a pivotal role in regulating chromatin architecture and function. These PTMs include acetylation, methylation, phosphorylation, ubiquitination, SUMOylation, and proline isomerization [48]. They either activate or repress gene transcription depending on their specific context [49]. The enzymes involved in histone modification include histone acetyltransferases (HATs), deacetyltransferases (HDACs), histone methyltransferases (HMTs), histone demethylases (KDMs), kinases, ubiquitinating ligases, ADP-ribosyltransferases, SUMOylation enzymes, glycosylases, and biotinylases. In MSCs, these epigenetic modifications are critically involved in lineage commitment. MSCs possess the capacity to differentiate into osteoblasts, adipocytes, or chondrocytes, a process that requires precise coordination of lineage-specific transcriptional programs [50]. Increasing evidence indicates that MSC differentiation is accompanied by distinct changes in histone modification patterns, which serve to either activate or repress key transcription factors that drive development toward specific cell fates.

Histone acetylation is mediated by acetyltransferases (HAT) which transfer the acetyl group to lysine residues in the N-terminal tails of histone H3 and H4 [51]. HATs can modify nuclear histones, which serve as the structural scaffold for DNA organization and constitute the fundamental repeating units of chromatin [52]. By adding acetyl groups to the lysine residues of histones, HATs neutralize the positive charge of these residues, weakening their electrostatic interaction with negatively charged DNA. This modification results in chromatin decondensation, facilitating enhanced accessibility of transcription factors and regulatory proteins to DNA [53].

Persistent histone acetylation induces severe nuclear abnormalities in human MSCs, ultimately promoting nuclear damage and cellular senescence [54]. CBP (CREB-Binding Protein) and p300 (E1A-binding protein) are closely related transcriptional co-activators [55]. CBP/p300 possesses HAT activity and its acetylation of histones neutralizes the positive charge of lysine residues, increasing DNA accessibility for transcription factors. This epigenetic mechanism regulates diverse biological processes, including cell proliferation, differentiation, and survival, cell cycle control, DNA repair, cellular stress response, and metabolic regulation [56].

Notably, CBP/p300-mediated acetylation can also stabilize HIF-1α and enhances FOXO3 activity, which synergistically upregulates BNIP3 expression under hypoxic conditions [57]. This pathway further enhances apoptosis resistance, migratory capacity, and lipid metabolism in WJ-SCs [58].

Histone deacetylases (HDACs) are an important class of epigenetic regulators that remove acetyl groups from histones, thereby compacting chromatin structure and suppressing gene transcription. Interestingly, HDACs regulate self-renewal of MSCs by modulating the expression levels of anti-aging polycomb group genes (PcGs) and the senescence-mediating jumonji-domain-containing 3 (JMJD3). Their activity influences cellular senescence through interactions with microRNAs and high-mobility-group A2 (HMGA2), which in turn alters histone modification patterns [59].

HDAC inhibition produces diverse effects in WJ-SCs. Specifically, HDAC1 inhibition increases acetylation of histone H4 (H4K5Ace), activating genes involved in endodermal and hepatic differentiation in WJ-SCs [60]. It also upregulates cardiac-specific genes, including GATA4, NKX2.5, and MLC (myosin light chain) [61], and hepatic makers (*FOXA2*, *CK8*, AFP, and TAT) [62]. However, TSA (tri-chostatin) treated WJ-SCs exhibit limited hepatic functionality compared to mature hepatocytes [63]. Interestingly, HDAC inhibition alone fails to differentiate WJ-SCs into pancreatic endocrine-like cells, likely due to the requirement for concurrent DNA demethylation of pancreatic lineage-specific gene promoters [64].

**Figure 1:**
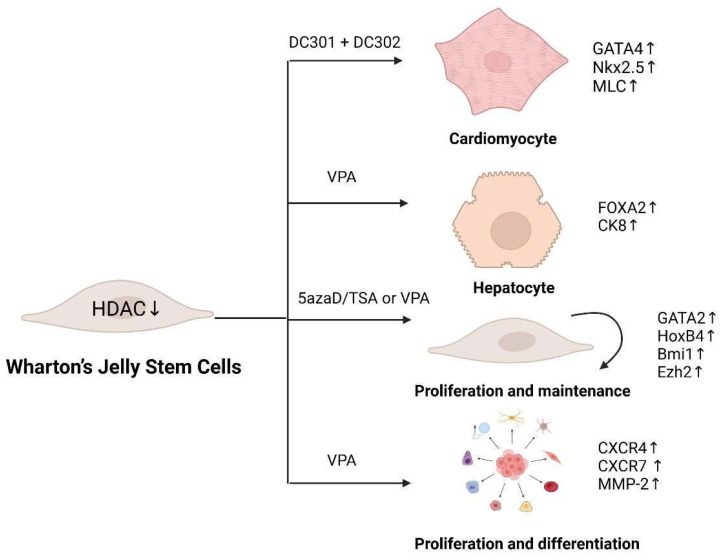
Different HDAC inhibitors regulate WJ-SC differentiation.

HDAC inhibitors also modulate WJ-SC self-renewal by transient DNA demethylation and histone acetylation, activating genes such as *GATA2*, *HOXB4*, *BMI1*, and *EZH2*, thereby promoting WJ expansion or maintenance [65]. Valproic acid (VPA), a specific HDAC inhibitor, enhances WJ-SC migration, proliferation, and differentiation by upregulating CXCR4, CXCR7, and MMP-2, promoting tissue repair [66]. However, the use of VAP reduces *HIST1H3C* expression, potentially compromising pluripotency [67]. While VPA and sodium butyrate suppress adipogenic, chondrogenic, and neurogenic differentiation, they enhance osteogenic potential [68]. The functions of diverse HADC inhibitors in WJ-SCs are summarized in Figure 1 (List of Abbreviations can be found in Supplemental Table S1).

Histone methylation is a complex and highly specific epigenetic modification that involves adding one or more methyl groups (methylation modifications) to lysine (Lys) or arginine (Arg) residues of histones. This process is catalyzed by histone methyltransferases (HMTs) using S-adenosylmethionine (SAM) as the methyl donor [69]. Methylation can occur at lysine residues by monomethylation (me1), bimethylation (me2) and trimethylation (me3), or at arginine residues through monomethylation (me1) and symmetric or asymmetric dimethylation (me2s and me2a) [70]. The biological function of histone methylation depends on the location and type of modification. For example, histone H3K4me3 is commonly associated with active gene transcription [71], whereas histone H3K27me3 is associated with gene silencing [72]. Histone methylation is catalyzed by specific histone methyltransferases that can alter the structure of chromatin and facilitate the binding of transcription factors and other regulatory proteins, thereby enhancing the transcriptional activity of genes [73]. For instance, the H3K4me3 modification exhibits cell type and gene specific distribution patterns, marking not only promoter regions but also extending into gene bodies to fine-tune expression levels. In undifferentiated WJ-SCs, the promoter regions of the key osteogenic transcription factors RUNX2/p57 and SP7 lack active histone marks, including H3 acetylation (H3ac), H3K27ac, and H3K4me3. Upon osteogenic induction, RUNX2/p57 expression was robustly activated, coinciding with acquisition of active marks (H3ac, H3K27ac, H3K4me3) in its P1 promoter region. On the other hand, SP7 remained transcriptionally silent due to persistent repressive marks (H3K9me3, H3K27me3) and the presence of H3K4me1 in its promoter [74].

Histone demethylation is a key epigenetic regulatory process that dynamically controls methylation of histone tails through specific families of demethylases, such as the KDM (lysine-specific demethylase) family [75]. These enzymes employ JmjC-domain-containing catalytic centers to oxidatively remove methyl groups from histones in an FAD (flavin adenine dinucleotide)- and oxygen-dependent manner, restoring lysine or arginine residues to their unmethylated state [76]. For instance, the histone demethylase KDM6B mainly demethylates histone H3K27, eliminating mono- or bis-methyl tags to activate gene expression [77]. DM6B promotes osteogenic differentiation, suggesting therapeutic potential for periodontitis treatment [78]. JARID1B can remove activating H3K4me3 marks from the *RUNX2/P57* P1 promoter, thereby suppressing RUNX2 expression in undifferentiated WJ-MSCs and inhibiting osteogenic differentiation [79]. KDM3A and KDM4C (H3K9-specific demethylases) play a role in heterochromatin reorganization by activating the transcription of cohesin complex components NCAPD2 and NCAPG2. This process is critical for limiting the DNA damage response and cellular senescence in WJ-SCs. Inhibition of KDM3A/4C increases DNA damage and accelerates senescence, while their overexpression—or direct NCAPD2 activation—enhances heterochromatin stability and genome protection [80].

### 2.3. Non-Coding RNA

Non-coding RNA (ncRNA) refers to RNA molecules that do not code for proteins but can modulate cellular function and play a role in regulation. ncRNAs include several types, with the primary ones being: micro ribonucleic acids (miRNAs), which are approximately 21–25 nucleotides long and can inhibit translation or promote degradation by binding to target mRNAs [81]. Long non-coding RNAs (lncRNAs), which are more than 200 nucleotides long, are involved in chromatin remodeling, transcriptional and post-transcriptional regulation [82]. Small interfering RNAs (siRNAs) can cause the degradation of specific mRNAs through RNA interference mechanisms [83]. Piwi interacting RNAs (piRNAs) play a role in animal germ cells and are mainly responsible for transposon silencing and genome stability [84]. Lastly, Circular RNAs (circRNAs), which have a closed-loop structure, act as miRNA sponges to regulate gene expression [85].

MicroRNAs (miRNAs) regulate gene expression via binding to target mRNAs either locally or through microvesicle-mediated transport, acting as post-transcriptional repressors or occasional activators [86]. These small non-coding RNAs modulate critical cellular processes including differentiation, survival, and apoptosis by interfering with the translation of target messenger RNAs (mRNAs) [87]. The degree of complementarity between the seed site and the target 3′-UTR determines the regulatory outcome: perfect complementarity induces mRNA degradation, while imperfect pairing blocks translation.

It has been discovered that overexpression of miR-196a-5p enhances alkaline phosphatase (ALP) activity, mineralization, and the expression of osteogenic marker genes in WJ-SCs, suggesting that miR-196a-5p promotes WJ-SCs’ osteogenic differentiation at the cellular level [88]. Similarly, overexpression of miR-424 significantly increases Osteocalcin protein secretion and promotes WJ-SCs’ osteogenic differentiation [89]. The Sonic hedgehog (Shh) signaling pathway is crucial in skeletal development, homeostasis maintenance and disease progression. Interestingly, miR-342-3p reduces the expression of Sufu through the N-terminal activation of the Shh ligand in the Shh signaling pathway, promoting WJ-SCs’ osteogenic differentiation and bone regeneration [90]. Additionally, miRNAs can interact with histone modification-related enzymes to regulate cell differentiation. For instance, miR146A acts as a negative regulator of JMJD3, and the downregulation of miR146A and upregulation of JMJD3 occur during osteogenic differentiation of WJ-SCs [91].

In terms of neuronal differentiation, upregulation of miR-1290, miR-26b, miR-194, and miR-124a, along with downregulation of miR-4521 and miR-543, promote the expression of the neuronal marker MAP2 [92]. Overexpression of miR-34a in WJ-derived neural precursor cells inhibits the cellular motility and properties associated with oxidative phosphorylation, electron transport, and ATP synthesis related to neural precursor gene expression [93].

In the context of WJ liver differentiation, several miRNAs were significantly upregulated. The target genes of these miRNAs are involved in inhibiting several processes of liver differentiation, such as epithelial–mesenchymal transition (EMT) and TGF-β receptor signaling pathway. Conversely, significantly downregulated miRNAs, whose target genes are involved in multiple processes promoting liver differentiation, such as liver development and Wnt signaling pathway [94]. In addition, increased expression of WJ hepatocyte-specific miRNA after VPA pretreatment contributed to enhancing the efficiency of WJ liver differentiation [95].

CXCL12 and SIKE1 were identified as direct target genes of miR-146a-5p. CXCL12 is a chemokine that induces the migration of MSCs [96], while SIKE1 is a repressor that regulates NF-kB through an atypical pathway [97]. Decreased expression of miR-146a-5p inhibited the proliferation but enhanced migration of WJ-SCs [98]. Under hypoxic conditions, miRNA-145 could inhibit TGF-β signaling by targeting TGF-β receptor II (TGFβRII), promoting WJ differentiation into type II alveolar epithelium instead of fibroblast-like cells [99]. In addition, miR-218 overexpression increased the expression of CD34, CD45, CD133, and c-Kit in WJ-SCs, and upregulated the expression of HoxB4 and NF-Yα in conjunction with microphthalmia-associated transcription factor (MITF), thereby inducing the differentiation of WJ-SCs into hematopoietic stem cells [100].

**Figure 2:**
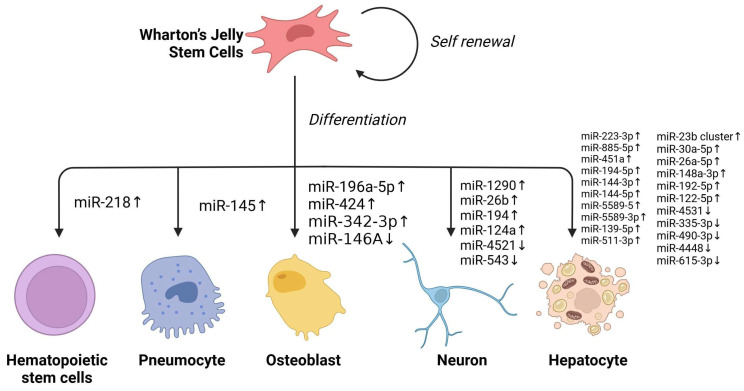
Effects of different miRNA regulation patterns on WJ differentiation.

Though there has been significant progress on the versatile roles of miRNAs in WJ-SCs (Figure 2), more effort should be put into comprehending the importance of miRNAs in terms of gene regulation and biological functions.

Extracellular vesicles (EVs) are membrane-bound particles secreted by cells and act as carriers for various molecules, including miRNAs, thus facilitating intercellular communication [101]. EVs can protect and sort miRNAs, and their detection, thus offering a promising avenue for disease diagnosis [102]. It is noteworthy that WJ-SCs-derived EVs have gained significant attention due to their regenerative, immunomodulatory, and anti-inflammatory properties. MSCs-derived EVs can facilitate wound healing by activating Wnt/β-catenin signaling [103] and improve cardiac tissue repair by reducing fibrosis and cardiomyocyte apoptosis via Smad7 regulation [104]. MSCs-EVs also possess potent immunomodulatory properties, making them promising candidates for autoimmune and inflammatory diseases, including rheumatoid arthritis (RA) [105], Graft-versus-Host Disease (GVHD) [106], and inflammatory bowel disease (IBD) [107]. Further, MSCs-EVs hold great therapeutic potential in other diseases such as liver fibrosis [108], osteoarthritis [109], and liver cancer [110]. These studies strongly suggest that functional miRNAs can be carried by WJ-EVs and utilized as a promising cell-free therapeutic tool with applications in regenerative medicine, immunomodulation, and disease treatment [111]. Future studies should focus on clinical translation and large-scale production to harness their full potential.

Compared to protein-coding mRNAs, lncRNAs have a lower average abundance, generally reside in the nucleus, and possess greater tissue specificity with lower interspecies sequence conservation. lncRNAs can regulate cellular physiological activities by controlling nuclear structure and transcription, regulating telomere stability, and directing epigenetic modification complexes to targeted genomic loci [112]. Their working mechanisms include base pairing interactions with mRNAs to override mRNA splice sites in the nucleus or miRNA binding sites in the cytoplasm; base pairing with miRNAs to deplete miRNAs; base pairing with cytoplasmic mRNAs to degrade or protect mRNAs; and also linking transcriptional complexes to regulate cellular transcription [113].

In terms of WJ-SC differentiation to germ-like cells, ZEB2-AS1 (also known as Zeb2NAT), a lncRNA, is associated with the epithelial–mesenchymal transition (EMT) process [114], and its up-regulation promotes the differentiation of WJ-SCs to germ-like cells related to the expression of ZEB2-AS1. Increased expression of TMEVPG1 (also known as IFNG-AS1) and HOTTIP lncRNAs promotes the differentiation of WJ-SCs to sperm cells. The LncRNA GAS5, once considered an oncogene, was found to be associated with the survival of female germline stem cells [115], and its expression was decreased during WJ-SC differentiation into spermatocytes [116]. When WJ-SCs were induced to differentiate into pancreatic islet cells, HI-LNC12, HI-LNC15, and HI-LNC71 were associated with the promotion of differentiation into β progenitor cells lncRNA AC009014.3, Gs1-72m21.1, and CTBP1-AS2 were linked with the developmental regulation of pancreatic β-cells; lncRNA: XLOC_050969, LINC00883, XLOC_050981, XLOC_050925, MAP3K14-AS1, RP11-148K1.12 and CTD2020K17.3 were involved in the regulation of insulin secretion in pancreatic β-cells [117]. However, overexpression of lncRNA H19 promotes chondrogenic differentiation in WJ [118].

Circular RNAs (circRNAs) are a unique class of endogenous ncRNAs that form closed continuous loops by reverse splicing with covalently attached 3′ and 5′ ends. They can act as microRNA sponges to mediate gene expression [119]. For example, CircFOXP1 can target miRNAs such as miR-17-3p and miR-127-5p to promote the proliferation and differentiation of WJ-SCs [120]. circRNAs can also regulate cellular functions through the circRNA-miRNA-mRNA network. For instance, during the repair of damaged endometrium using WJ-SCs, circRNA hsa_circRNA_0111659 acts as a miRNA sponge and competitively binds to miR-17-5p, miR-20b-5p, and miR-93-5p, releasing the target VEGF mRNA to regulate the repair process [121]. Additionally, the WJ-derived exosome circHIPK3 has been found to protect high glucose-treated human umbilical vein endothelial cells via the miR-20b-5p/Nrf2/VEGFA axis [122]. Furthermore, circRNA_05432, circRNA_08441, and circRNA_01536 play key roles in the differentiation of WJ-SCs into cardiomyocyte-induced cells [123]. CDR1as is involved in maintaining WJ proliferation and differentiation. Knockdown of *CDR1as* leads to the downregulation of the expression of stemness transcription factors (STFs) resulting in impaired adipogenic and osteogenic potential [124].

### 2.4. RNA Modification

RNA modification is the process of adding or removing specific chemical groups to an RNA molecule to regulate RNA function, stability, localization, and translational efficiency [125]. Some of the most common modifications include N6-methyladenosine (m^6^A), which is added by methylation enzymes (e.g., METTL3 and METTL14) and removed by demethylation enzymes (e.g., FTO and ALKBH5). This modification is involved in regulating mRNA stability, translational efficiency, and splicing [126]. Other common RNA modifications include 5-methylcytidine (m^5^C), pseudouridine (Ψ), and N1-methyladenosine (m^1^A), which exert a variety of biological functions by affecting the structure of RNAs and their interactions with proteins. RNA modifications play an important role in cellular differentiation, development, and response to stress, and are closely associated with the development of many diseases.

m^6^A is the most prevalent internal modification in mammalian RNAs. This modification process is precisely regulated by m^6^A writers, erasers, and readers. The m^6^A modification process is mediated by m^6^A writers, a class of large methyltransferase complexes consisting of METTL3, METTL14, and the nephroblastoma 1-associated protein (WTAP), which mediate the methylation of m^6^A on the adenosine of the target RNAs [127], a dynamic and reversible biological process [128]. Both METTL3 and METTL14 proteins contain methyltransferase structural domains. However, METTL3 is the catalytically active subunit that transfers methyl group to RNA. It binds independently to chromatin and localizes to the transcriptional start sites of active genes. whereas METTL14 plays both structural and non-catalytic roles in substrate recognition, thereby maintaining complex integrity and binding to substrate RNA [129]. Interestingly, recent studies have shown that a significant increase in m^6^A levels of circCTTN can promote osteogenic differentiation WJ-SCs. circCTTN does not interact directly with METTL3 but may contribute to this increased methylation through the interaction of the nucleolin 2 (NOP2) protein with the m^6^A-reading protein EIF3A [130]. Additionally, lncCCKAR5 can act as a scaffold to promote the interaction between MKRN2 and LMNA (a key regulator of cytoskeletal function and autophagy), thereby forming the lncCCKAR5/LMNA/MKRN2 complex to promote ubiquitin-mediated LMNA degradation and WJ-SC apoptosis [131]. Furthermore, miR615-3p inhibits osteogenic differentiation of WJ-SCs by targeting the 3′ untranslated region of *FBLN1* mRNA and interacting with YTHDF2 in an m^6^A modification-dependent manner [132].

Pseudouridine (Ψ), an isomer of uridine, is an abundant RNA modification that affects the function of tRNA and rRNA in translation, small nuclear RNA (snRNA) in splicing, and plays a role in pre-mRNA splicing, RNA stabilization, protein translation, and the cellular stress response [133]. And H/ACA snoRNA can direct the pseudouridylation of rRNA. The levels of specific H/ACA snoRNA change dynamically during the differentiation of stem cells into other cell types [134]. For example, H/ACA Box small nucleolar RNA 7A (SNORA7A), which is abundant in undifferentiated WJ-SCs, can enhance the self-renewal capacity of WJ-SCs by promoting the formation of the small nucleolar ribonucleoprotein complex (snoRNP), while inhibiting their osteogenic and adipogenic differentiation [135].

**Figure 3:**
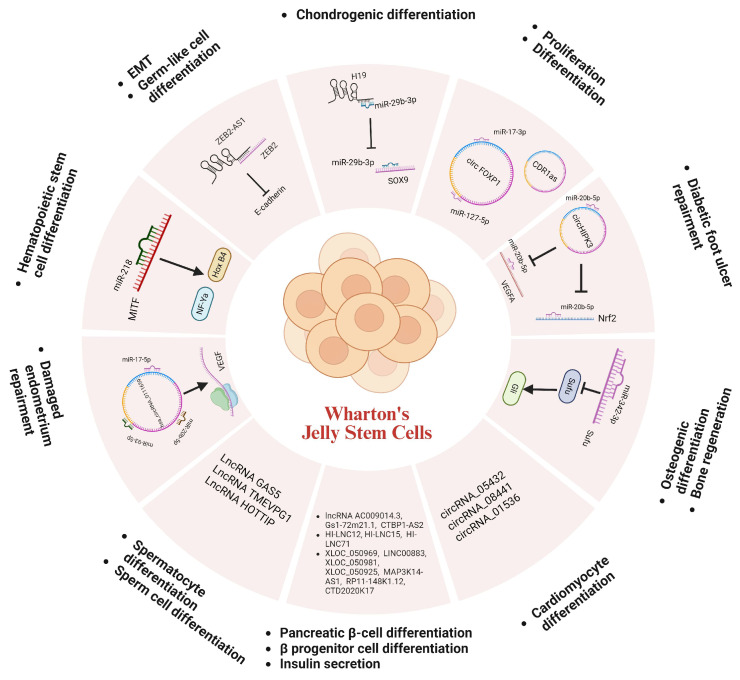
Functional non-coding RNAs and RNA modifications in WJ-SCs.

Collectively, ncRNAs, which include microRNAs (miRNAs), long non-coding RNAs (lncRNAs), and circular RNAs (circRNAs), regulate gene expression post-transcriptionally and are essential for functions of WJ-SCs. RNA modifications, including m^6^A, m^5^C, and pseudouridine (Ψ), dynamically regulate WJ-SC function by altering RNA stability, splicing, and translation. Both ncRNAs and RNA modifications play crucial roles in regulating the functions of WJ-SCs by influencing gene expression, stem cell maintenance, differentiation, and therapeutic potential (Figure 3).

**Figure 4:**
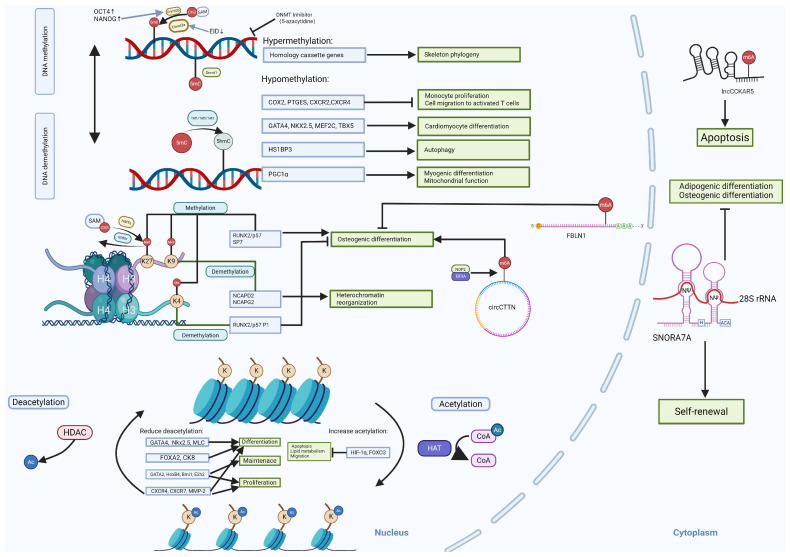
Diagram of epigenetic regulation in WJ-SCs.

In summary, epigenetic regulation plays a crucial role in stemness maintenance, differentiation, and the therapeutic efficacy of WJ-SCs. This review explores the key epigenetic mechanisms—DNA methylation, histone modifications, and non-coding RNAs—that govern WJ-SC behavior and their implications in regenerative medicine (Figure 4).

## 3. Conclusions and Prospects

Epigenetic mechanisms regulate gene expression without altering the DNA sequence, primarily through DNA methylation, histone modifications, and non-coding RNAs. DNA methylation, an early discovered epigenetic mark, suppresses gene transcription by adding methyl groups to DNA. Histone modifications, such as acetylation, methylation, and phosphorylation, can affect chromatin structure by altering the chemical state of histones, thereby controlling gene activity. Non-coding RNAs, especially miRNAs and lncRNAs, regulate gene expressions post-transcriptionally and at the chromatin level by interacting with mRNAs or chromatin.

The study of epigenetic mechanisms is particularly crucial in the field of WJ-SCs which are a popular research subject in regenerative medicine and tissue engineering due to their unique multi-potency and immunoregulatory properties. Continued advances in epigenetic engineering will enhance their clinical translation in regenerative and personalized medicine. While significant progress has been made, several emerging areas warrant further investigation. Firstly, the heterogeneity of WJ-SCs can be masked by bulk sequencing, single cell RNA-seq and single cell ATAC-seq can be carried out to link epigenetic states with gene expression. The multi-omics integration could reveal novel regulatory mechanisms in stemness, differentiation, and immunomodulation of WJ-SCs. Secondly, our current knowledge on functions of non-coding RNAs and RNA modifications in WJ-SCs is still limited. Future studies can focus on functional studies on lncRNAs/circRNAs and the systematic profiling of all types of RNA modifications in WJ-MSCs. These studies may uncover new therapeutic targets for enhancing the regenerative capacity of WJ-SCs. Thirdly, WJ-SCs may retain epigenetic memory from their tissue of origin. Hence, epigenetic priming or reprogramming strategies may be explored to enhance the therapeutic efficacy of WJ-SCs. Finally, one of the future directions may aim to engineer “tailored” WJ-SCs to enhance immunomodulation properties via epigenetic methods. These advances will deepen our understanding of WJ-SC biology and unlock their full potential in regenerative medicine.

## Figures and Tables

**Table 1:** Comparison of WT-SCs with other types of stem cells.

Feature	WJ-SCs	ESCs	iPSCs	BM-MSCs	AD-SCs
**Source**	Umbilical cord	Blastocyst	Reprogrammed cells	Bone marrow	Fat tissue
**Potency**	Multipotent	Pluripotent	Pluripotent	Multipotent	Multipotent
**Ethical Issues**	No	Yes	No	No	No
**Tumor Risk**	Low	High	High	Low	Low
**Immunogenicity**	Low (HLA-G+)	High	Patient-matched	Moderate	Moderate
**Proliferation**	High	Very High	High	Moderate	Moderate

Note: WJ-SCs: Wharton’s Jelly stem cells; ESCs: embryonic stem cells; iPSCs: induced pluripotent stem cells; BM-MSCs: bone marrow mesenchymal stem cells; AD-SCs: adipose-derived stem cells.

## Supplementary Materials

The following supporting information can be downloaded at: https://www.mdpi.com/article/10.3390/ijms26157169/s1.
